# Frontal Fibrosing Alopecia and Reproductive Health: Assessing the Role of Sex Hormones in Disease Development

**DOI:** 10.3390/jpm14010072

**Published:** 2024-01-05

**Authors:** Alexandra-Maria Roman, Răzvan-Cosmin Petca, Mihai Cristian Dumitrașcu, Aida Petca, Andreea-Iuliana Ionescu (Miron), Florica Șandru

**Affiliations:** 1 Dermatology Department, “Elias” University Emergency Hospital, 011461 Bucharest, Romania; alexandra-maria.roman@rez.umfcd.ro (A.-M.R.); florica.sandru@umfcd.ro (F.Ș.); 2 Department of Urology, “Carol Davila” University of Medicine and Pharmacy, 020021 Bucharest, Romania; 3 Department of Urology, “Prof. Dr. Th. Burghele” Clinical Hospital, 050659 Bucharest, Romania; 4 Department of Obstetrics and Gynecology, “Carol Davila” University of Medicine and Pharmacy, 020021 Bucharest, Romania; 5 Department of Obstetrics and Gynecology, University Emergency Hospital of Bucharest, 050098 Bucharest, Romania; 6 Department of Obstetrics and Gynecology, “Elias” Emergency University Hospital, 011461 Bucharest, Romania; 7 Department of Oncological Radiotherapy and Medical Imaging, “Carol Davila” University of Medicine and Pharmacy, 020021 Bucharest, Romania; 8 Department of Medical Oncology, Colțea Clinical Hospital, 030167 Bucharest, Romania; 9 Department of Dermatovenerology, “Carol Davila” University of Medicine and Pharmacy, 020021 Bucharest, Romania

**Keywords:** Frontal Fibrosing Alopecia, menopause, contraceptives, hormonal imbalances

## Abstract

Frontal Fibrosing Alopecia (FFA) is a distinctive form of cicatricial alopecia characterized by gradual hairline recession, predominantly affecting postmenopausal individuals, thus implying a potential hormonal origin. This narrative review, spanning 2000 to 2023, delves into PubMed literature, focusing on the menopausal and hormonal status of women with FFA. The objective is to unravel the intricate nature of FFA and its plausible associations with hormonal dysregulations in women. While menopause remains a pivotal demographic characteristic linked to FFA, existing data suggest that its hormonal imbalances may not fully account for the development of FFA. Conversely, substantial evidence indicates a strong association between a reduction in fertile years, particularly through surgical interventions leading to an abrupt hormonal imbalance, and FFA in women. Additionally, exposure to hormone replacement therapy or oral contraceptives has shown varying degrees of association with FFA. Gynecologists should maintain a heightened awareness regarding the ramifications of their interventions and their pivotal role in overseeing women’s fertility, recognizing the potential influence on the progression of FFA. The recurrent theme of hormonal disruption strongly implies a causal connection between alterations in sex hormones and FFA in women. Nevertheless, this relationship’s extent and underlying mechanisms remain subjects of ongoing debate.

## 1. Introduction

Frontal Fibrosing Alopecia (FFA) is a patterned lymphocytic primary cicatricial alopecia. Although it was deemed rare initially, it has now become the foremost cause of cicatricial alopecia globally [1,2]. FFA exhibits a distinct clinical presentation featuring symmetrical frontotemporal hairline recession and bilateral eyebrow loss; however, patterns associated with varying degrees of body hair loss have been recently identified [3]. In consequence, FFA has a marked adverse impact on the patient’s health-related quality of life, including their psychological and emotional well-being [4,5]. FFA emerged as a novel dermatological pathology in 1994 and was initially termed postmenopausal fibrosing alopecia due to its predominant occurrence in postmenopausal women, suggesting a potential hormonal origin. This fact was reinforced in 2003 when the first two cases of FFA following bilateral oophorectomy were reported [6,7]. 

Genetic vulnerability to FFA is also evident, as human leucocyte antigen (HLA) profiling studies have identified two susceptible haplotypes (C17:01:01:02/B42:01:01 and C07:02:01:03/B07:02:01:01) in familial cases profiling studies [8]. A second study supports the genetic etiology by demonstrating that a high percentage (66%) of FFA patients have both a personal history and a first-degree family history of autoimmune disease [9]. Moreover, hypothyroidism due to autoimmune thyroiditis is associated with up to 30% of patients with FFA [10]. These proposed pathogenesis theories support the idea that the manifestation of the disease within families can be attributed to a shared environmental trigger that may be exacerbated by an inherited susceptibility [9,11]. 

Numerous studies have attempted to establish a connection between FFA and various lifestyle triggers. Regular use of facial sunscreen is one of the most contentious environmental factors associated with FFA. FFA patients often exhibit high sunscreen usage, possibly due to the common inclusion of sunscreens in facial skincare products, leading to increased exposure [12,13]. On the other hand, one of the studies included in our review also reported the statistical association of FFA with exposure to organic, alkyl phenolic compounds (OR = 1.48; 95% CI 1.05–2.08) which are also found in cosmetic products. These organic compounds have estrogen-disruptive capacity and the ability to inhibit the transformation of dehydroepiandrosterone [14]. Remarkably, statistically significant associations with FFA have been identified in relation to dietary choices and smoking, as documented in the literature [15,16]. Last but not least, exposure to exogenous hormones and therapies that interfere with hormonal homeostasis may play a pivotal role in disease development, which will be further detailed below.

On the other hand, the role of hormones in the development of FFA has been the subject of research and hypothesis ever since it was first described, and such, correlations between FFA and four genetic loci linked to the CYP1B1 gene mutation have been found through a genome-wide association study. The CYP1B1 gene is widely expressed and encodes the cytochrome P4501B1 microsomal enzyme also referred to as exogenous monooxygenase. By using the oxidative metabolism process, this enzyme changes estradiol and estrogen into the corresponding hydroxylated catechol estrogen; this suggests that a direct cause of FFA development may be the increased exposure of females to CYP1B1 substrates [17]. The hormonal imbalance theory has been so compelling that, despite finding no significant differences, hormonal profiles were examined through androgen receptor and estrogen beta receptors (ERβ) in both clinically affected and unaffected scalp FFA patients [18]. 

Menopause warrants particular attention due to its strong association with FFA. It marks the loss of reproductive function brought on by the total depletion of the limited supply of ovarian follicles. The declining number of ovarian follicles signals the onset of menopause, which is characterized by variations in reproductive hormones, particularly the decline in the secretion of the hormones estrogen and progesterone, and alterations in the menstrual cycle; these changes are impacted by sociocultural, psychological, and ethnic variables [19,20]. These hormonal changes during menopause have a major effect on the skin and its appendages because of the prevalence of estrogen and, to a lesser extent, progesterone receptors in the dermis and epidermis. Menopause has been associated with several different scenarios involving hair loss. Still, it is currently challenging to accurately understand the physiological changes in hair that follow menopause, the relationship between these changes, and the changes’ long-term prognosis. Since it can be challenging to determine which hair changes call for medical attention due to this relative lack of knowledge, various studies began looking into the patterns of postmenopausal hair changes. According to one report, the most common type of scalp hair loss was diffuse scalp hair loss, which was reported by 26% of women and was found to be significantly correlated with both body hair loss and aging (*p* < 0.05). On the other hand, frontal hair loss was reported by 9% of women, and it was linked to both a younger age and higher facial hair scores (*p* < 0.05) [21]. Of the alopecias caused by decreased ovarian activity, androgenic alopecia is arguably the most well known. The increased effect of androgens on the sebaceous glands and hair follicles as a result of the decrease in progesterone is thought to be partially responsible for this phenomenon. Androgens lead to miniaturization of the hair follicles situated on the scalp while having the opposite effect of transforming vellus hair into terminal hair in other androgen-dependent areas, thus generating hirsutism. Investigations should be conducted into postmenopausal women who have recently experienced rapid onset hirsutism or hair loss, as these symptoms could indicate a more serious underlying condition, such as virilizing tumors that dramatically raise androgen levels. Furthermore, the decrease in estrogen levels leads to the shortening of the anagen phase of the hair follicle cycle during menopause, which can also determine diffuse hair loss [22,23,24,25].

We aim to analyze the influence of hormonal interventions on the risk of development of Frontal Fibrosing Alopecia.

## 2. Materials and Methods

A PubMed literature search was conducted using the terms: “Frontal Fibrosing Alopecia” and “menopause” (or “estrogen” or “progesterone”), from 2000 to 2023, amassing 67 search results. We followed different types of data that reference the hormonal status of patients with FFA and included only clinically relevant studies (excluded case reports or series with less than 5 cases) that provided enough information on the dermatological issues in relation to sex hormone imbalances. We included a total of 23 original studies in our analysis, consisting of 16 retrospective studies and 7 prospective studies. Among the prospective studies, one is an observational cross-sectional study, while the rest are case-control studies. Our analysis comprises a total of 2667 patients diagnosed with Frontal Fibrosing Alopecia (FFA), with 2572 of them being female.

## 3. Results

### 3.1. Hormonal Particularities of FFA Female Patients

Although Frontal Fibrosing Alopecia (FFA) has historically been more prevalent in postmenopausal women, it is noteworthy that documented cases also include premenopausal women and male patients with FFA [26,27,28,29,30,31]. Despite these variations, the perimenopausal hormonal status hypothesis remains one of the strongest when it comes to FFA causality. 

One study that examined this hypothesis was conducted by Bernárdez et al. on 43 premenopausal women diagnosed with FFA with a median age of 42.5 years. After analyzing the laboratory results of parameters such as Follicle-Stimulating Hormone (FSH), Luteinizing hormone (LH), Estradiol, Androstenedione, 17-Hydroxyprogesterone (17-OH-P), Prolactin, Dehydroepiandrosterone sulfate (DHEA-s), and Testosterone, they determined that only 9% of these forty-three premenopausal patients had analytic alterations suggestive of perimenopause, while the remaining 39 (91%) had the sex hormone profiles expected of fertile women. Thus, it was concluded that serum sex hormone levels may not be directly linked to the pathophysiology of FFA, as they are not consistently altered in premenopausal FFA patients [32] (Table 1).

Furthermore, a case-control study involving forty women whose age and menstrual status matched revealed that the FFA case group of menstruating women had significantly lower FSH levels than the control group of menstruating women (*p* = 0.03). The postmenopausal groups did not exhibit the same phenomenon (*p* = 0.91). In addition, FSH (*p* = 0.04) and LH (*p* = 0.04) levels were lower in postmenopausal patients with premenopausal onset of FFA than in those with postmenopausal onset of disease. However, the same study reported that without considering their menstrual statuses, there was no significant difference between the sex hormone levels in the case and control groups. Likewise, there was no other statistically significant difference between the two groups’ hormonal levels among the postmenopausal women. Additionally, it was shown that the duration since FFA onset had no effect on hormonal levels, except for the serum level of free testosterone, which was found to decrease in patients overall as the disease’s duration increased (correlation coefficient: −0.53, *p* = 0.02). However, this correlation was not significant in the postmenopausal and menstruating women subgroups separately. As a result, the study also concluded that serum sexual hormone levels do not appear to have a direct correlation with the pathophysiology of FFA [33].

In contrast, a study consisting of 490 FFA cases showcased that hormonal status in female patients was mainly normal. However, different correlations between disease activity and disease onset could be demonstrated. While the Lichen Planopilaris Activity Index Scoring System objectively quantifies disease activity in LPP and has been utilized for assessing FFA activity, alternative and perhaps more accurate scoring systems are also available for evaluating FFA activity [34,35]. Abnormal values of estrogen (r = −0.664) and testosterone (r = 0.462) were highly correlated with LPPAI scores. Abnormal estrogen values were also associated with age at onset of symptoms (r = 0.34). In contrast, abnormal sex hormone binding globulin (SHBG) values were correlated with menopause (r = −0.458), abnormal TPO/TRAb values (r = 0.471), and current hormonal contraception (r = 0.693). The small number of patients with abnormal hormonal values and the failure to distinguish between excess and deficiency when recording abnormal hormone values provide limitations to the interpretation of these associations. Nonetheless, the findings’ internal validity was reinforced by the finding that aberrant estrogen levels were linked to unusual testosterone levels [36].

Nonetheless, a recently discussed theory in the literature, the low androgen level theory, serves as the foundation for a new and developing concept [37] During a study on hormonal and endocrine dysfunction in patients with various forms of lichen planopilaris, Ranasinghe et al. found that androgen excess/polycystic ovary syndrome was the most common dysfunction in all LPP subtypes. In contrast, for FFA patients, the primary hormonal disturbance was low androgens, particularly low DHEA and DHEAS [38]. These are the most prevalent circulating steroid hormones in humans. According to Mendoza-Milla et al., women’s production of DHEA and DHEAS peaks between the ages of 25 and 30 and begins to decline at 60, reaching only 10% to 20% of the previous peak levels, meaning that at menopause, when FFA occurs, these hormones’ levels are decreased [39]. 

Besides their effect on fibrosis pathways through their actions on the PPAR γ pathway, DHEA and DHEAS have numerous regulatory effects on the immune system and fat metabolism [39,40,41]. DHEA exhibits a potent antifibrotic effect on fibroblasts, impacting their migration, survival, proliferation, and differentiation while diminishing collagen production. Certain fibrotic diseases, like idiopathic pulmonary fibrosis, have been linked to lower DHEAS levels, as well as many autoimmune diseases, such as systemic lupus erythematosus and pemphigus [42,43,44]. The reduced levels in DHEAS and FFA seem to be linked through the role of DHEAS in suppressing fibrosis and TGF-β1. DHEA regulates the function of PPAR-c, which is a negative regulator of fibrotic events triggered by TGFβ1. A decrease in DHEA and androgens may cause the pro-fibrotic condition in FFA [45]. In 2020, a different case-control study found that FFA patients had significantly lower DHEAS and androstenedione serum levels than controls (*p*-value = 0.038 and 0.012, respectively). Free testosterone, LH, FSH, 17-OH progesterone, and prolactin serum levels did not significantly differ between the FFA group and the control group [46]. 

**Table 1:** Hormonal particularities of FFA female patients.

First Author, Year	Type of Study	Studied Population	Sex Hormone Statuses
Kanti 2019 [36]	Observational, cross-sectional study	N = 490 FFA patients N1 = 467 (95%) female FFA patients N2 = 23 (5%) male patients with FFA	N1 = 33 (88%) Testosterone (T)values within normal range N2 = 28 (96%) DHEAS values normal in tested women N3 = 26 (92%) SHBG values normal in tested women N4 = 0 abnormal PRL values N5 = 0 abnormal free androgen index
Nasiri 2020 [46]	Case-control study	N1= 30 women with FFA N2 = 34 healthy age and menopausal status matched controls	N1 = 12.93 PRL (ng/mL) median in FFA patients N2 = 11.69 PRL (ng/mL) median in controls *p* = 0.882 N3 = 10.24 Luteinizing hormone (LH) (mIU/mL) median in FFA patients N4 = 11.69 LH (mIU/mL) median in controls *p* = 0.619 N5 = 24.21 FSH (mIU/mL) median in FFA patients N6 = 27.72 FSH (mIU/mL) median in controls *p* = 0.288 N7 = 0.12 17-Hydroxyprogesterone (17-OH-prog) (ng/mL) median in FFA patients N8 = 0.36 17-OH-prog (ng/mL) median in controls *p* = 0.275 N9 = 0.97 Free T (pg/mL) median in FFA patients N10 = 1.4 Free T (pg/mL) median in controls *p* = 0.135 N11 = 79.26 DHEA-S (µg/dL) median in FFA patients N12 = 152.34 DHEA-S (µg/dL) median in controls *p* = 0.038 N13 = 1.41 Androstenedione (A4) (ng/mL) median in FFA patients N14 = 2.31 A4(ng/mL) median in controls *p* = 0.012
Sasannia 2020 [33]	Case-control retrospective study	N1 = 20 women with FFA (mean age of 46.9 years) N2 = 20 healthy controls (mean age of 47.20 years)	N1 = 6.40 Mean LH (IU/L) in FFA cases N2 = 9.72 Mean LH (IU/L) in controls *p* = 0.52 N3 = 9.00 Mean FSH (IU/L) in FFA cases N4 = 15.11 Mean FSH (IU/L) in controls *p* = 0.03 N5 = 19.88 Mean PRL (ng/mL) in FFA cases N6 = 20.63 Mean PRL (ng/mL) in controls *p* = 0.44 N7 = 0.42 Mean Total T (ng/mL) in FFA cases N8 = 0.40 Mean Total T (ng/mL) in controls *p* = 0.58 N9 = 1.28 Mean Free T (pg/mL) in FFA cases N10 = 1.57 Mean Free T (pg/mL) in controls *p* = 0.48 N11 = 123.87 Mean DHEAS (µg/dL) in FFA cases N12 = 172.75 Mean DHEAS (µg/dL) in controls *p* = 0.2 N13 = 38.09 Mean LH (IU/L) in Postmenopausal at onset FFA cases N14 = 19.70 Mean LH (IU/L) in Cyclic at onset FFA cases N15 = 55.27 Mean FSH (IU/L) in Postmenopausal at onset FFA cases N16 = 36.72 Mean FSH (IU/L) in Cyclic at onset FFA cases N17 = 11.68 Mean PRL (ng/mL) in Postmenopausal at onset FFA cases N18 = 6.40 Mean PRL (ng/mL) in Cyclic at onset FFA cases N19 = 0.35 Mean Total T (ng/mL) in Postmenopausal at onset FFA cases N20 = 0.31 Mean Total T (ng/mL) in Cyclic at onset FFA cases N21 = 0.84 Mean Free T (pg/mL) in Postmenopausal at onset FFA cases N22 = 0.72 Mean Free T (pg/mL) in Cyclic at onset FFA cases N23 = 52.66 Mean DHEAS (µg/dL) in Postmenopausal at onset FFA cases N24 = 71.00 Mean DHEAS (µg/dL) in Cyclic at onset FFA cases
Bernárdez 2017 [32]	Retrospective study	N = 43 premenopausal women with FFA N1 = 42.5 years, median age	N1 = 91% FSH within normal range N2 = 87% LH within normal range N3 = 82% Estradiol within normal range N4 = 97% A4 within normal range N5 = 90% 17-OH-P within normal range N6 = 90% PRL within normal range N7 = 100% DHEA-s within normal range N8 = 92% T within normal range N9 = 39 (91%) had hormone profiles expected of fertile women N10 = 4 (9%) had alterations suggestive of perimenopause
Ranasinghe 2017 [38]	Retrospective study	N = 53 women with FFA	N1 = 9 (17.0%) androgen excess/PCOS N2 = 17 (32.1%) low androgens N3 = 9 (17.0%) low/high androgens N4 = 2 (3.8%) hirsute/low androgens N5 = 9 (17.0%) ovarian cyst only N6 = 2 (3.8%) Ovarian cyst/low T

### 3.2. The Effect of Fertile Years and Early Menopause on the Development of FFA

A series of other authors herein cited also reinforced the theory that most women (60–95,0%) are postmenopausal at the onset of FFA, having a median age of symptom onset between 50.9 and 62 years [28,47,48,49,50,51,52,53] (Table 2). While most researchers predicted this result, Buendía-Castaño showcased in 2018 that not only is FFA associated with menopause, but more importantly, that it is perhaps associated with a decrease in fertile years. In the aforementioned case-control study, it was demonstrated that FFA patients enter menopause 2 years earlier than their healthy counterparts (47.7 years, respectively 49.7 years, *p* = 0.01) and have 1.7 years less fertile life than the controls (34.8 years, respectively 36.5 years, *p* = 0.004). Additionally, it has been estimated that the risk of developing FFA increases by 7% each year of advance in menopause, thus indicating that a longer fertile life might be a protective factor regarding FFA [54]. On the other hand, reproductive life itself seemed to enhance the risk of alopecia, a history of pregnancy having been linked to the appearance of FFA in a case-control study (OR = 1.6; 95% CI 1.06–2.41). Furthermore, one of the largest studies in the field reported that 14% of females had an early menopause (<45 years) and had a 49 years (range 23–60) mean age of menopause, despite having the onset of FFA at a mean age of 56 years (range 21–81) [55].

Moreover, because FFA is considered by many authors a particular form of lichen planopilaris (LPP), Meinhard conducted a comparative study that underlined the fact that although both pathologies appear later in life, the proportion of postmenopausal women was significantly higher in the FFA group than in patients with classic LPP (95.5%, respectively, 53.6%, *p* = 0.025), also demonstrating that patients with FFA were significantly more likely (*p* = 0.001) to be postmenopausal than were patients with classic LLP [56]. 

Furthermore, fertile status seemed to affect the extension of the disease to other areas. Grassi et al. emphasized that eyelash involvement was limited to postmenopausal women, while in a study by Starace et al. on 65 FFA patients, the percentages of axillary and body hair involvement were higher in premenopausal women [48,52].

**Table 2:** Fertile status in women with FFA.

First Author, Year	Type of Study	Studied Population	Fertile Statuses
Banka 2014 [49]	Retrospective study	N = 62 patients with FFA N1 = 61 females	Menopausal N = 49 (80%) Premenopausal N = 12 (20%)
Grassi 2021 [48]	Retrospective study	N = 119 patients with FFA N1 = 8 men N2 = 111 female	N1 = 101, (91.0%) Menopausal at onset of FFA N2 = 10 (9.0%) Premenopausal at onset of FFA N3 = 50.9 years mean age at menopause onset
Buendía-Castaño 2018 [54]	Case-control study	N1 = 104 female FFA patients N2 = 208 age-matched controls	N1 = 47.7 age of menopause for FFA patients N2 = 49.7 years, age of menopause for controls *p* = 0.01 N3 = 34.8 years of fertile life for FFA patients N4 = 36.5 years of fertile life for the control group *p* = 0.004 N5 = 94 (91.3%) postmenopausal FFA patients N6 = 189 (90.9%) postmenopausal controls
Panchaprateep 2020 [57]	Retro-prospective cohort study	N = 58 patients with FFA	N1 = 53 (91.4%) Menopause status at present N2 = 16 (27.6%) Onset at pre-menopause N3 = 48 (46–51) Mean age of menopause N4= 20 (37.7%) Surgical menopause
Imhof 2018 [50]	Retrospective study	N = 148 female FFA patients	N1 = 57,4 mean age at onset of symptoms N2 = 129 (87.2%) postmenopausal at presentation N3 = 14 (9.4%) premenopausal at presentation N4 = 5 (3.4%) menopause status undisclosed. N5 = 48.9 years mean age of menopause
Tan 2009 [58]	Retrospective study	N1 = 18 patients with FFA	N1 = 15 (83%) menopausal N2 = 3 (17%) premenopausal N3 = 55.5 mean age of onset (range 34–71)
Müller Ramos 2021 [51]	Case-control study	N1 = 451 FFA patients N2 = 451 sex-matched controls N3 = 434 (96%) females N4 = 17 (4%) males	N1 = 272 (60%) menopausal FFA patients N2 = 200 (44%) menopausal controls N3 = 47 years mean age of disease onset near menopause
Vañó-Galván 2013 [55]	Retrospective study	N = 355 patients with FFA N1= 343 women with FFA N2= 12 men with FFA N3 = 61 years mean age (range 23–86)	N1 = 294 menopausal women N2 = 49 premenopausal women N3 = 49 years (range 23–60) mean age of menopause N4 = 49 (14%) females with early menopause (<45 years) N4.1 = 31(9%) surgical menopause N5 = 56 years (range 21–81) mean age of onset of FFA
Starace 2019 [52]	Case-control study	N = 65 females with FFA N2 = 62.5 years (range 42–87) mean age	N1 = 57 (87.75%) menopausal N2 = 8 (12.25) premenopausal N3 = 51.5 years (43–61 years) mean age at menopause onset N4 = 6 (10.5%) had developed premature menopause (≤45 years) N4.1 = 3 (4.6%) had hysterectomy N5 = 9 (13.8%) reported prolonged of irregular menses N6 = 10.3 years average time between the onset of menopause and the development of FFA
Conde Fernandes 2011 [53]	Retrospective study	N = 11 women with FFA N1 = 64.9 years mean age	N1 = 10 (90.9%) postmenopausal women N2 = 1 (0.1%) premenopausal woman
Mervis 2019 [59]	Retrospective study	N = 91 patients with FFA N1 = 87 women N2 = 4 men N3 = 59.6 years mean age	N1 = 30 (34%) premenopausal at first visit
Dlova 2013 [60]	Retrospective study	N= 20 patients with FFA N1 = 19(95%) female N2 = 1 (5%) male N3 = 42 yrs mean age of onset	N1 = 14 (73%) premenopausal N2 = 4 (27%) menopausal
Tosti 2005 [61]	Retrospective study	N = 14 women with FFA N1 = 62 years mean age (range 54 and 78 years)	N1 = 14 (100%) menopausal N2 = 5.5 yeas mean time (range 2–12 years) from menopause to disease onset
Kanti 2019 [36]	Observational, cross-sectional study	N = 490 FFA patients N1 = 467 (95%) female FFA patients N2 = 23 (5%) male patients with FFA N3 = 60 years mean age of onset (IQR 53–68 years)	N1 = 84% women were postmenopausal
Moreno-Arrones 2019 [14]	Case-Control study	N1 = 578 women N2 = 289 women with FFA N3 = 289 female controls N4 = 77 men N5 = 19 men with FFA N6 = 58 male controls	N1 = 34.2 (17–48) number of reproductive years-controls N2 = 34.5 (15–52) number of reproductive years-FFA *p* = 0.59 N3 = 219 (75.8%) controls who had pregnancies N4= 241 (83.4%) cases who had pregnancies *p* = 0.03
Nasiri 2020 [46]	Case-control study	N1= 30 women with FFA N2 = 34 healthy age and menopausal status matched controls N3 = 51.07 ± 9.22 years–mean age of FFA patients N4 = 51.15 ± 8.16 years–mean age of controls	N1 = 15 (50.0%) patients with FFA were postmenopausal N2 = 15 (50.0%) patients with FFA were premenopausal N3 = 19(55.9%) controls were postmenopausal N4 = 15(44.1%) controls were premenopausal
MacDonald 2012 [28]	Retrospective study	N = 60 women with FFA	N1 = 3 (5%) premenopausal N2 = 55 (95%) menopausal
Suchonwanit 2020 [62]	Retrospective study	N = 56 patients with FFA N1 = 54 (96.4%) women with FFA N2 = 2(3.6%) men with FFA N3 = 51 years–average age of disease onset (range, 39–80 years)	N1 = 48 (88.9%) postmenopausal women N2 = 8 (11.1%) premenopausal women
Heppt 2018 [47]	Retrospective study	N = 72 FFA patients N1 = 70 (97.2%) women N2 = 2 (2.8%) men	N1 = 57 (81.4%) Postmenopausal N2 = 6 (8.6%) Premenopausal N3 = 7 (10.0%) Unknown menopausal status
Sasannia 2020 [33]	Case-control retrospective study	N1 = 20 women with FFA (mean age of 46.9 years) N2 = 20 healthy controls (mean age of 47.20 years)	N1 = 8 participants were postmenopausal in each group N2 = 12 participants were cyclic in each group N3 = 6 presented the disease after the onset of menopause
Bernárdez 2017 [32]	Retrospective study	N = 43 premenopausal women with FFA N1 = 42.5 years, median age	N = 43 (100%) premenopausal
Meinhard 2014 [56]	Retrospective study	N1 = 31 women with FFA N2 = 1 man with FFA	N = 95.5% postmenopausal

### 3.3. Surgical Menopause and FFA

In 2009, Tan and colleagues carried out a study on eighteen FFA female patients, two of whom had hysterectomies. They remarked that the surgery, which was conducive to surgical menopause, had been performed prior to the onset of Frontal Fibrosing Alopecia in both cases, supporting the previously mentioned theory of early onset menopause being a trigger for FFA [58] (Table 3). Similarly, Suchonwanit et al. reported in 2020 that although only a small percentage (3.7%) of the studied group have had hysterectomies and oophorectomies, FFA did not begin to develop in either of these patients before the surgically induced menopause [62].

Hysterectomies are one of the most common gynecological procedures done worldwide. It has been highlighted that this surgery is combined with a unilateral or bilateral oophorectomy in 60% of women who underwent laparoscopic procedures and 68% who had an abdominal hysterectomy [63]. On the bright side, recently, it has been reported that oophorectomy rates have generally declined over the previous 20 years [64].

Iatrogenic menopause, also known as surgical menopause, results from the removal of both ovaries prior to the physiological decline in ovarian function. Unlike natural menopause, which occurs gradually, surgical menopause is linked to an abrupt decrease in ovarian sex steroid production [65,66,67,68]. Hormone replacement therapy (HRT) has been strongly recommended in women who have undergone bilateral oophorectomy before the natural age of menopause, at least until the estimated physiological age of menopause [69,70,71,72]. 

Furthermore, Vañó-Galván et al. carried out one of the largest studies on the topic of FFA, involving 355 patients. They discovered that a significant percentage of women with FFA are presenting with early menopause (<45 years) or have had hysterectomies (13%), particularly in the premenopausal period, with 9% of the subjects having had surgical menopause [55]. Starace et al. also reiterated that 10.5% of the FFA patients in their study group had developed premature menopause, but in this case, only 4.6% had hysterectomies [52]. On the other hand, a study conducted on 148 FFA revealed a markedly elevated prevalence of surgically induced menopause. Approximately 39.6% of the cohort had undergone gynecologic surgical interventions. This subset consisted of 26 individuals (18.7%) who had experienced premenopausal total hysterectomy and 18 individuals (13%) who had undergone premenopausal oophorectomy [50].

Meanwhile, there are studies that report a much higher rate of iatrogenic menopause (37.7%) [57], while a case-control study also highlighted that the frequency of hysterectomies is significantly higher in FFA patients than in healthy controls (OR 2.14 [95% CI 1.35–3.39], *p* = 0.002) [54]. In contrast, one of the largest multicenter case-control studies reported no statistically significant difference in hysterectomy and oophorectomy prevalence between FFA women and controls(*p* = 1.00, respectively *p* = 0.15) [14].

### 3.4. Hormone Replacement Therapy and FFA

Hormone replacement therapy (HRT) aims to correct the hormonal loss that happens during the menopausal transition through supplementation. It is indicated for the treatment of vasomotor symptoms of menopause and genitourinary syndrome of menopause, as well as for osteoporosis prophylaxis. Conventional HRT mimics the hormones produced by the human ovary by including an estrogen and progesterone component [73]. Beyond controlling moderate to severe menopausal symptoms, hormone replacement therapy (HRT) also lowers the risk of fractures and type 2 diabetes. Furthermore, given that this patient population is at an elevated lifetime risk for several chronic disorders that can be mitigated with timely initiation of hormone replacement therapy, HRT should be prioritized in otherwise healthy women who experience early or premature menopause, even in the absence of bothersome symptoms. Acknowledging the potential risks linked to HRT is crucial, but so is appreciating the advantages of treatment [74,75,76,77].

Hormone replacement therapy has long been regarded as a parameter that requires monitoring in relation to FFA, as evidenced by the studies covered in our review. Meinhard et al. proved that patients with FFA are significantly (32.3% vs. 11.9%, *p* = 0.025) more likely to be on HRT than patients with classic LPP. However, the fact that FFA patients experience menopause at a higher rate than LPP patients constrained the study and may have contributed to the previously mentioned outcome [56] Another study that supports the correlation of HRT with FFA onset is the one published in 2019 by Moreno-Arrones et al., which described a statistically significant difference between FFA patients who took HRT and healthy controls who took HRT (OR = 1.76; 95% CI 1.11–2.8 *p* = 0.02) [14] Tosti et al. also reported that 35.7% of FFA patients were undergoing estrogen replacement therapy at the onset of the disease. In contrast, Mervis et al. described that only 13% of women were undergoing either hormonal birth control or HRT at the time of the FFA diagnosis [59,61].

While other publications reveal an important rate of FFA patients with a history of HRT [62], in a large study conducted by Kanti in 2019, no association was found between hormone replacement therapy and FFA age of onset, disease activity, or symptoms [36].

Because of the controversies of the effect of HRT on the appearance of FFA, many studies investigating the hormonal status of FFA patients decided to consider HRT one of the exclusion criteria, besides other endocrinological dysfunctions, hormonal contraception, and steroid supplement use [46] (Table 4).

### 3.5. Contraceptive Measures and FFA

The intricate relationship between hormones and the skin means that as hormonal contraceptive therapies develop and become more widely used, dermatologists and gynecologists must be aware of their mechanisms and potential effects on the skin.

The most widely prescribed type of contraceptive pill is the combined oral contraceptive (COC) pill, which contains both estrogen and progesterone [78].

The dermatologic effects of hormonal contraceptives are due to their capacity to affect androgen receptors through progestins, as well as estrogen receptors through mestranol or ethinylestradiol [79]. Although COCs are the most well-known type of hormonal contraceptives, hormonal intrauterine devices (IUDs) have become more and more common in recent years. They are long-acting, effective contraceptives that work by releasing levonorgestrel into the uterus with no major impact on long-term fertility [80].

While high-index androgen contraceptives have been incriminated in the past for triggering androgenic alopecias, there is also evidence that hormonal contraceptives, particularly COCs could improve androgenic alopecia, particularly through the estrogen component, that promotes hair growth [81,82,83,84]. Moreover, hormonal IUDs are also reported to have an estimated cumulative incidence of alopecia in 0.33% of patients [85].

Overall, research indicates that progesterone-only treatments, like hormonal IUDs and implants, often cause or exacerbate a variety of dermatological illnesses, including alopecia and rosacea, which are commonly linked to FFA. The role of hormonal contraceptives in alopecia is less well-established, but research in these areas is ongoing [86].

Consequently, a large case-control study conducted by Buendía-Castaño identified IUD use as a protective factor regarding FFA (OR 0.22 [95% CI 0.06–0.84], *p* = 0.027). However, they also found no statistically significant difference between the two groups’ use of oral contraceptives, a result that has been reinforced in the Kanti et al. case-control study, which described no correlation between COC use and disease activity or age of onset [36,54] In contrast, large retrospective studies focusing solely on the FFA population described an important rate of association (26.6–51%) of former contraceptive pill use in these patients [50,57] (Table 5).

### 3.6. Gynecologic Neoplasias and Tamoxifen Use in Relation to FFA

Since progesterone and estrogen have a major impact on normal breast, ovarian, and uterine development, they are also the hormones most commonly associated with breast and ovarian cancers, affecting their genesis, metastasis, and prognosis [87,88,89]. Selective estrogen receptor modulators (SERMs) like Tamoxifen and Raloxifen have been successfully used to treat patients at every stage of estrogen receptor-positive breast cancer. These substances have a potent antiestrogenic effect on breast tissue while maintaining an estrogen-like effect when it comes to bone metabolism [90,91,92].

A vast majority of studies herein described considered a probable association between FFA and these two neoplasias because of the hormonal link and the implications of the treatments they necessitate (Table 6). Despite many studies having reported a low grade of association between tamoxifen use for breast cancer and FFA (3–4.2% of FFA patients had undergone Tamoxifen therapy), Buendía-Castaño listed Tamoxifen use as a significant risk factor for developing FFA in a large case-control study (OR 14.89 [95% CI 2.42–91.68], *p* = 0.004) [47,49,50,54]. On the other hand, a different case-control study conducted by Moreno-Arrones reported no significant statistical difference between FFA patients and healthy controls in what ovarian cancer, breast cancer, and Tamoxifen use was concerned (*p* = 0.14, respectively, *p* = 0.52, *p* = 0.76), although they reported a significant association of Raloxifen therapy and FFA (*p* = 0.03) [14].

## 4. Discussion

The studies herein presented support the hypothesis that a significant number of patients with FFA are subjected to a series of hormonal disruptions. Sex steroid hormones are thought to have a role in the development of FFA, particularly because of the frequent onset of FFA in postmenopausal women, similar patterning and co-existence with androgenic alopecia, and a reportedly good response to 5α-reductase inhibitors and other antiandrogenic drugs. Multiple studies support antiandrogenic therapy for patients with FFA, claiming they aid with disease progression and, in some cases, even partial regrowth. Finasteride’s success in treating certain patients may also suggest that androgens have a role in the disease’s etiology [26,93,94,95,96]. On the other hand, there is a hypothesis suggesting that the abnormal decrease in DHEA during postmenopausal stages, which typically stimulates PPARc (peroxisome proliferator-activated receptor gamma), might play a role in the development of Frontal Fibrosing Alopecia [45].

However, as seen before, many studies could not prove a correlation between serum levels in sex hormones and disease onset or progression. This might also be due to the fact that a solid genetic background is usually needed to develop the disease. A study conducted on 233 FFA patients upheld this by identifying that the vast majority of FFA patients (75.2%) did not have the protective rs1800440 polymorphism in CYP1B1. Furthermore, it also reported that 83.8% of the subjects carried the rs9258883 polymorphism in HLA-B*07:02, which is involved in a deviant immune response, thus the genetic component is further demonstrated [97]. CYP1B1 selectively catalyzes the 4-hydroxylation of estradiol and is highly expressed in estrogen target tissues, such as the ovary, uterus, and mammary glands. The novel discovery that estradiol regulates human CYP1B1 through estrogen implies that estrogen’s physiological control over the CYP enzymes involved in estrogen metabolism would be important for maintaining the homeostasis of estrogens in specific organs [98]. These results could generate further research in the field and eventually might provide a therapeutic option targeting FFA’s genetic element. The aforementioned genetic study also recorded clinical and demographic data that further highlighted the high rate of hysterectomies and oophorectomies at an early age (mean age 45.2 years), as well as the high prevalence of oral contraceptives (36.9%) and HRT (14.9%). These data support the theory that a reduced fertile life and advancement in menopause are strongly linked to FFA.

Without a doubt, menopause remains the most significant demographic characteristic when it comes to FFA. During the perimenopausal phase, or the transition to menopause, there are noticeable drops in hair density and diameter. After menopause, estrogen levels drop sharply, but androgen secretion gradually decreases with age and is sustained until later in life by elevated LH production. The decrease in estrogens is accompanied by a marked reduction in SHBG concentrations and an increase in the free androgen index due to the tendency of adipose tissue to accumulate, while insulin resistance and hyperinsulinemia may augment androgen secretion after menopause. Testosterone and dihydrotestosterone determine a regression in the scalp hair follicles, a process that is the key factor in androgenic alopecia. This phenomenon is diminished by progesterone, which has been shown to inhibit 5-α reductase, thereby decreasing dihydrotestosterone production. However, the gradual decline in the body hair score with aging implies that changes in scalp hair are not exclusively associated with the endocrine shifts associated with menopause [99,100,101,102]. This might be also possible since the skin is a factory for synthesizing numerous chemicals, most of which have autocrine or paracrine functions, in addition to being affected by various hormones [103].

A significant association between FFA and androgen deficiency is reported throughout multiple studies. As the risk of FFA increases with the advancement in menopause, which in turn correlates with lower androgen levels, this might be a plausible pathogenical hypothesis. A phenomenon that might support the hypoandrogenic theory is the progressively larger number of FFA that have begun to be diagnosed in male patients who had received hormone-interfering therapies. In a pivotal 2014 study, a patient undergoing neoadjuvant hormonal therapy for prostate cancer developed FFA 8 years after initiating estrogen treatment [49]. Tolkachjov et al. also reported that a significant part of male FFA patients had hypogonadism despite normal total testosterone levels. This was probably due to their history of using phosphodiesterase 5 inhibitors for erectile dysfunction [31]. A broader study revealed that 61.5% of male FFA patients exhibited abnormal sex hormone levels, with high 17-OH and SHBG levels being prevalent deviations [30]. In a 2022 study, male FFA patients with a history of antiandrogenic medications for prostate cancer treatment were studied, revealing a connection between these medications and FFA onset. These medications induced secondary hypogonadism by lowering testosterone levels, and 7.7% of patients also had high SHBG levels [104].

## 5. Conclusions

In essence, Frontal Fibrosing Alopecia (FFA) exhibits a robust correlation with fluctuations in sex hormones, being particularly triggered by the ones happening during surgical and physiological menopause. Consequently, it is advisable to undertake thorough hormonal assessments and a comprehensive examination of medical history, specifically focusing on hormonal therapies, gynecologic surgeries, and hormone-dependent neoplasia, when confronted with an FFA patient. Serologic investigations assume importance, particularly in cases where patients are obese or manifest other clinical indicators of hormonal imbalance, such as hirsutism in women. These investigations offer a pathway to a more integrated understanding of the role played by these factors in the genesis of Frontal Fibrosing Alopecia. Elucidating these causal relationships holds the potential to unveil targeted therapeutic approaches, thereby substantially enhancing the quality of life for individuals profoundly impacted by this ailment. Furthermore, additional research is imperative to thoroughly establish the extent to which the aforementioned factors contribute to the onset of FFA.

## Figures and Tables

**Table 3:** Gynecologic surgery in women with FFA.

First Author, Year	Type of Study	Studied Population	Gynecologic Surgery
Buendía-Castaño 2018 [54]	Case-control study	N1 = 104 female ffa patients N2 = 208 age-matched controls	N1 = 30 (28.8%) FFA patients with hysterectomy N2 = 28 (13.5%) controls with hysterectomy OR 2.14 [95% CI 1.35–3.39], *p* = 0.002)
Panchaprateep 2020 [57]	Retro-prospective cohort study	N = 58 patients with FFA	N1 = 20 (34.5%) Hysterectomy
Imhof 2018 [50]	Retrospective study	N = 148 female ffa patients	N1 = 55 (39.6%) hysterectomy, of the patients 139 who disclosed hysterectomy status N2 = 26 (18.7%) premenopausal total hysterectomy N3 = 18 (13%) premenopausal oophorectomy
Tan 2009 [58]	Retrospective study	N1 = 18 patients with FFA	N1 = 2 had hysterectomies
Vañó-Galván 2013 [55]	Retrospective study	N = 355 patients with FFA N1 = 343 women with FFA N2 = 12 men with FFA	N1 = 46 (13%) hysterectomy N2 = 31 premenopausal hysterectomies N3 = 15 postmenopausal hysterectomies
Moreno-Arrones 2019 [14]	Case-Control study	N1 = 578 women N2 = 289 women with FFA N3 = 289 female controls N4 = 77 men N5 = 19 men with FFA N6 = 58 male controls	N1 = 36 (12.5%) controls with Hysterectomy N2 = 36 (12.5%) cases with Hysterectomy *p* = 1.00 N3 = 22 (7.6%) controls with oophorectomy N4 = 32 (11.1%) cases with oophorectomy *p* = 0.15
Suchonwanit 2020 [62]	Retrospective study	N = 56 patients with FFA N1 = 54 (96.4%) women with FFA N2 = 2(3.6%) men with FFA N3 = 51 years–average age of disease onset (range 39–80 years)	N1 = 2 (3.7%) hysterectomy and oophorectomy and neither of these patients had a premenopausal onset of FFA
Starace 2019 [52]	Case-control study	N1 = 65 females with FFA N2 = 62.5 years (range 42–87) mean age	N1 = 3 (4.6%) had hysterectomy

**Table 4:** Hormone replacement therapy in women with FFA.

First Author, Year	Type of Study	Studied Population	Hormone Replacement Therapy Use
Banka 2014 [49]	Retrospective study	N = 62 patients with FFA N1 = 1 male N2 = 61 females	N1 = 3 (5%) Hormone replacement therapy N2 = 4 (6%) Estrogen therapy (including one male patient, which does not constitute HRT) N3 = 2 (3%) Progesterone therapy
Panchaprateep 2020 [57]	Retro-prospective cohort study	N = 58 patients with FFA	N1 = 7 (12.1%) Received hormone replacement therapy
Imhof 2018 [50]	Retrospective study	N = 148 female FFA patients	N1 = 90 had hormone replacement therapy (HRT) (history documented in 90 patients) N2 = 57 (63.3%) history of HRT use in the form of systemic estrogen and/or systemic progesterone
Mervis 2019 [59]	Retrospective study	N = 91 patients with FFA N1 = 87 women N2 = 4 men N3 = 59.6 years mean age	N1 = 11 (13%) women either using hormonal birth control or HRT at the time of FFA diagnosis
Tosti 2005 [61]	Retrospective study	N = 14 women with FFA N1 = 62 years mean age (range 54 and 78 years)	N1 = 5 (35.7%) patients undergoing estrogen replacement therapy at debut
Kanti 2019 [36]	Observational, cross-sectional study	N = 490 FFA patients N1 = 467 (95%) female FFA patients N2 = 23 (5%) male patients with FFA N3 = 60 years mean age of onset of symptoms (IQR 53–68 years)	N1 = 22% had hormonal replacement therapy
Moreno-Arrones 2019 [14]	Case-control study	N1 = 578 women N2 = 289 women with FFA N3 = 289 female controls N4 = 77 men N5 = 19 men with FFA N6 = 58 male controls	N1 = 34 (11.8%) controls who used HRT N2 = 55 (19%) cases who used HRT *p* = 0.02
Suchonwanit 2020 [62]	Retrospective study	N = 56 patients with FFA N1 = 54 (96.4%) women with FFA N2 = 2 (3.6%) men with FFA N3 = 51 years–average age of disease onset (range, 39–80 years)	N1 = 7 (12.9%) received HRT
Meinhard 2014 [56]	Retrospective study	N1 = 31 women with FFA N2 = 1 man with FFA	N1 = 10 (32.3%) had HRT

**Table 5:** Contraceptive measures in women with FFA.

First Author, Year	Type of Study	Studied Population	Contraceptive Method
Buendía-Castaño 2018 [54]	Case-control study	N1 = 104 female FFA patients N2 = 208 age-matched controls	N1 = 5 (4.8%) FFA patients with use of IUD N2 = 30 (14.4%) controls with use of IUD OR 0.22 [95% CI 0.06–0.84], *p* = 0.027 N3 = 104 (43.3%) patients who took oral contraceptives N4 = 103 (49.5%,) controls who took oral contraceptives
Panchaprateep 2020 [57]	Retro-prospective cohort study	N = 58 patients with FFA	N1 = 16 (27.6%) history of taking oral contraceptive N2 = 3 (5.2%) history of intrauterine device
Imhof 2018 [50]	Retrospective study	N = 148 female FFA patients	N1 = 26 [51%] history of oral contraceptive pill use
Mervis 2019 [59]	Retrospective study	N = 91 patients with FFA N1 = 87 women N2 = 4 men N3 = 59.6 years mean age	N1 = 11 (13%) women either using hormonal birth control or HRT at the time of the FFA diagnosis
Kanti 2019 [36]	Observational, cross-sectional study	N = 490 FFA patients N1 = 467 (95%) female FFA patients N2 = 23 (5%) male FFA patients N3 = 60 years mean age of onset of symptoms (IQR 53–68 years)	N1 = 21% hormonal contraception
Moreno-Arrones 2019 [14]	Case-control study	N1 = 578 women N2 = 289 women with FFA N3 = 289 female controls N4 = 77 men (FFA + controls)	N1 = 143 (49.5%) controls who took oral contraceptives N2 = 141 (48.8%) cases who took oral contraceptives *p* = 0.86
Meinhard 2014 [56]	Retrospective study	N1 = 31 women with FFA N2 = 1 man with FFA	N1 = 1 (3.2%) took oral contraceptives

**Table 6:** Gynecologic neoplasia in women with FFA.

First Author, Year	Type of Study	Studied Population	Gynecologic Neoplasia and Antiestrogenic Therapies
Banka 2014 [49]	Retrospective study	N = 62 patients with FFA N1 = 1 male N2 = 61 females	N1 = 2 (3%) received Tamoxifen
Buendía-Castaño 2018 [54]	Case-control study	N1 = 104 female FFA patients N2 = 208 age-matched controls	N1 = 8 (7.7%) FFA patients with breast cancer N2 = 5 (2,4%) controls with breast cancer OR 3.20 [95% CI 1.07–9.54], *p* = 0.028) N3 = 7 (6.7%) FFA patients with use of tamoxifen N4 = 2 (0.1%) controls with use of tamoxifen OR 14.89 [95% CI 2.42–91.68], *p* = 0.004
Imhof 2018 [50]	Retrospective study	N = 148 female FFA patients	N1 = 6 (4.1%) had history of Tamoxifen use for breast cancer
Moreno-Arrones 2019 [14]	Case-control study	N1 = 578 women N2 = 289 women with FFA N3 = 289 female controls N4 = 77 men N5 = 19 men with FFA N6 = 58 male controls	N1 = 13 (4.5%) controls with breast cancer N2 = 10 (3.5%) cases with breast cancer *p* = 0.52 N3 = 2 (0.7%) controls who had ovarian cancer N4 = 0 cases who had ovarian cancer *p* = 0.15 N5 = 5 (1.7%) controls who took Tamoxifen N6 = 6 (2.1%) cases who took Tamoxifen *p* = 0.76 N7 = 0 controls who took Raloxifen N8 = 6 (2.1%) cases who took Raloxifen *p* = 0.03
Heppt 2018 [47]	Retrospective study	N = 72 FFA patients N1 = 70 (97.2%) women N2 = 2 (2.8%) men	N1 = 3 (4.2%) history of breast cancer

## Data Availability

Data sharing not applicable.
